# A novel endoscopic visible full-thickness cryoablation device on stomach

**DOI:** 10.1038/s41598-020-61595-x

**Published:** 2020-03-23

**Authors:** Wanwei Zheng, Yao Liu, Yujen Tseng, Jun Zhang, Wenshuai Li, Bangting Wang, Yida Pan, Jie Zhu, Zhongguang Luo, Feifei Luo, Jie Liu

**Affiliations:** Department of Digestive Diseases, Huashan Hospital, Fudan University, Shanghai, 200040 China

**Keywords:** Oesophagogastroscopy, Gastric cancer

## Abstract

Cryoablation has been used for the treatment of various sorts of solid visceral tumors, but few are reported on gastric tumor via endoscope, in terms of accurate control of ablation site, freezing depth and effective temperature. Thus, we developed a novel device, which could perform accurate cryoablation on the stomach via endoscope. This study aimed to evaluate the efficacy and safety of the device on porcine stomach. Results showed that the novel device could provide direct view of the operation space, allowing accurate and safe ablation of the stomach. Three minutes cryoablation caused a transmural, 1 cm radius gastric lesion. On serosal side, the temperature dropped to −64.2 °C, −34.1 °C, 26.1 °C at the center, 1 cm and 2 cm from center, respectively. Histopathology revealed acute ruptured cells with damaged glands in mucosa, partial disruption in muscularis propria and serosal slight exudation. Three months later, scar formed with complete recovery of gastric structure. No active bleeding or perforation of stomach, nor injury or adhesion of adjacent organs was observed. This endoscopic cryoablation device allowed safe, full-thickness cryoablation with effective temperature, which may provide an alternative treatment for gastric tumor.

## Introduction

Cryoablation, also known as cryosurgery, is the destruction or removal of living tissues through freezing in a controlled manner. Cryoablation was first used to treat uterine and breast cancers in the 1840s, before it gained the technological innovation that trocar-type probes, capable of delivering cooling source, equipped the cryoablation system in 1960s. Since then, the application of cryoablation was expanded to various sorts of tumor^1^. However, few cases report the use of endoscopic cryoablation on the treatment of gastric tumors, since this required more specialized equipment. Current reported endoscopic cryoablation devices are mainly spraying device, which are only appropriate for the treatment of mucosal lesion, such as Barrett’s esophagus^2–4^. These spraying devices, which are operated by spraying compressed carbon dioxide, can’t only cause transmural injury^3^. The depth that the lesion infiltrates is not sufficient to meet the demands for the treatment of gastric tumor^5^. It is difficult to ensure accurate cryoablation since the target is not fixed to the device and the vision is compromised when performing endoscopic spraying. Besides, risk of gastric perforation caused by excessive gastric distention is increased when cryogen gas dilates the stomach^6^. One reported contact cryoablation device is mainly used for the treatment of esophageal disease, which is composed of a high compliant balloon and cartridge containing liquid nitrous oxide. The balloon could expand and occupy the esophageal lumen, creating a circular cryoablation injury^7^. However, due to the large cavity and peristalsis of the stomach, it is difficult to control the volume of the balloon, thus the cryoablation could hardly be achieved on the stomach. Consequently, these reported endoscopic cryoablation devices do not apply to the treatment of gastric tumor, because they could not implement accurate cryoablation and efficient injury infiltration depth.

The present study introduces a novel cryoablation device, which consist of a cryoablation system and an endoscopic catheter with a closed balloon attached to its end. The device can pass through the forceps pipe of an electronic gastroscope.

The balloon can inflate to a fixed volume to help the operator carry out designated cryoablation, with stable supply and discharge of cryogen within the vacuum insulation catheter. *In vitro* study showed the balloon could form a regional temperature field as low as −120 °C in one minute. Based on the device, an animal experiment was designed to evaluate the efficacy and safety of the device.

## Materials and methods

### Cryoablation device

Cryoablation system (Shengjiekang, Ningbo, Zhejiang, China) is comprised of a cryoablation device (Fig. S1A) and an intraluminal cryoablation catheter. The cryoablation device works under a recirculation system, which can sustain stable liquid nitrogen and real-time recycle of cryogen under the working pressure of 23.8 atm, providing a vacuum insulation environment for the catheter. Cryoablation catheter (Fig. S1B) is composed of an 8 mm diameter balloon, catheter and handle. The balloon is deflated before passing through the 3.2 mm diameter forceps pipe of an electronic gastroscope (GIF-Q260J, Olympus, Tokyo, Japan). The balloon is inflated and then holds a fixed volume of 8 × 8 × 12 mm^3^ at the target gastric mucosa to carry out cryoablation. Experiments *in vitro* showed that the temperature of the balloon surface could reach −120 °C after 1 min in gelatin under the environment of 25 °C/33% RH, forming a temperature field of 1 cm in radius with temperature lower than −40 °C. The temperature would not further obviously decrease, nor the iceball expanded as cryoablation time extended after three minutes (Fig. S1C,D).

### *In vitro* experiments of cryoablation on 3D cell culture system

Before the *in vivo* experiment on porcine stomach, an *in vitro* experiment on matrigel was carried out to investigate the optimum cryoablation duration.

#### Cell culture

The human MKN-45 cell lines were purchased from the Cell Bank of Type Culture Collection of Chinese Academy of Sciences. The cells were cultured in DMEM (Gibco, USA) supplemented with 10% fetal bovine serum (FBS, Gibco), 50 IU/mL penicillin/streptomycin (Gibco), under 5% CO_2_ atmosphere at 37 °C. The exponentially growing cells were used for the following experiment. 1 × 10^7^ cells were mixed with 1.5 ml Matrigel (BD Bioscience), and then the mixture were seeded on a 3.5 cm culture dish to establish a 3D cell culture system, with a final width of 1.5 mm. The seeded cells were cultured for 12 hours before cryoablation.

#### Cryoablation

The cryoablation system was used to carry out cryoablation. The balloon was deflated and placed on the center of the 3D culture dish. One glove filled with 37 °C water was put under the culture dish to imitate the *in vivo* environment. Then the system started and perform 1 minute’, 3 minutes’ and 6 minutes’ cryoablation respectively, with 0 minute’s cryoablation as control (Fig. S1E).

**Figure 1:**
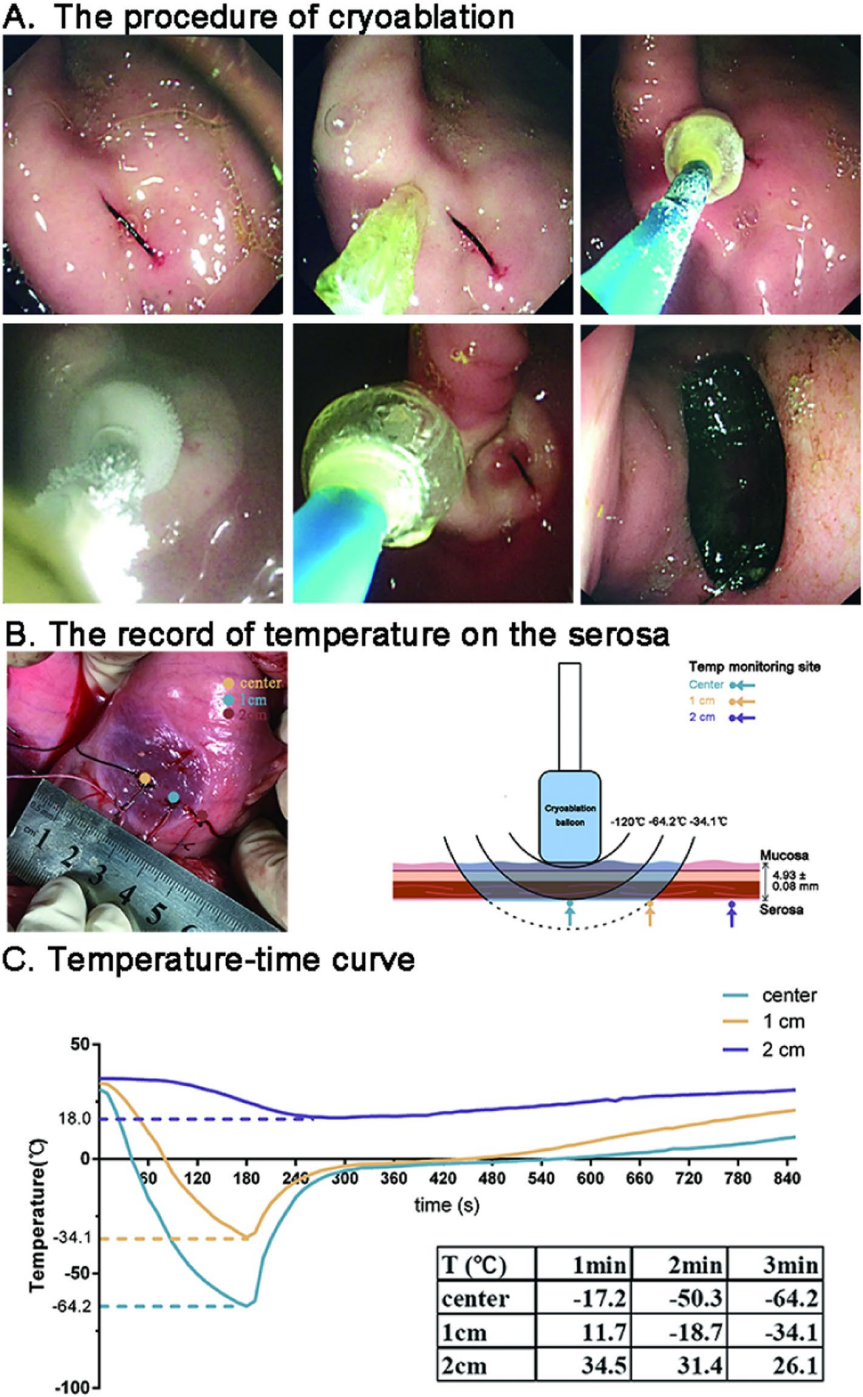
(**A**) The procedure of cryoablation. (**B**) Three thermometer probes were embedded on the gastric serosa side. The pattern illustrated the temperature monitoring site and circle temperature field caused by three minutes cryoablation. (**C**) The serosa temperature was recorded during the process of cryoabaltion and the temperature-time curve was illustrated.

#### Cell apoptosis assay

Cells on the center of the dish with an area of 12 mm*8 mm were harvested. Then, these cells were stained by Annexin V (BD, USA) for 30 min and followed by staining with 7-AAD (Invitrogen, USA) for 5 min. The percentage of apoptotic cells were measured by BD Calibur (BD).

### Animal studies

#### Ethics statement

Animal studies were approved by Ethical Committee of Department of Laboratory Animal Science of Fudan University (Approval NO. 201805002Z). All experimental procedures were performed according to ethical guidelines under protocols approved by the Ethical Committee of Department of Laboratory Animal Science of Fudan University.

#### Animal experiments

Endoscopy and ablation were performed on nine 5-month-old pigs (mean weight [±standard deviation] 46 ± 6 kg). The pigs were food-deprived 48 hours and water-deprived 12 hours prior to the procedure. Pre-anesthesia intramuscular injection of 3 mg/kg ketamine along with intravenous injection of 20 mg/kg pentobarbital sodium was administered. Subsequent intravenous injection of 6–8 mg/kg/h pentobarbital sodium was given to sustain anesthesia. The animals were placed in supine position with limbs fixed on the operation table. After intubation, mechanical ventilation was provided. Vital signs were monitored throughout operation. The nine pigs were sacrificed at different time points to observe cryoablation effects on different phase. Three pigs were sacrificed on the day of operation to observe acute phase injury (acute group, N = 3). Three pigs were sacrificed three weeks after operation to observe subacute phase change (subacute group, N = 3) and the last three were sacrificed three months after operation to observe chronic phase change (chronic group, N = 3).

#### The procedure of the cryoablation and the record of the cryoablation effects on experimental animals

The antrum, greater curvature side and lesser curvature side of gastric wall was selected to carry out cryoablation. After the upper endoscope was advanced into the stomach, the balloon was inserted to direct at the marked mucosa, and then the cryoablation system was started. Seconds later, the balloon expanded to a fixed volume to carry out cryoablation. After the balloon naturally flew off the gastric wall, the endoscope with balloon was withdrew. Re-examination by endoscopy was carried out 45 minutes after the cryoablation. Vital signs were monitored throughout the procedure and post-anesthetic care was performed. The pigs gradually resumed normal diet after one-day post-operative fasting. Muscular injection of 25 mg/kg/day cefazolin was given consecutively for three days to prevent infection. The pigs’ postoperative feeding and body weight was recorded. Euthanasia of the pigs in three groups was performed by injection of overdose pentobarbital sodium at the end of operation, three weeks later and three months later, respectively. After dissection, the stomach and adjacent structures were examined carefully.

#### Synchronous temperature record during the *in vivo* cryoablation operation

An abdominal median incision was made on one of nine pigs, exposing the stomach. After securing a target on the gastric antrum, a silk thread was sutured full-thickness from the gastric serosal side to mark the gastric mucosal side (Fig. 1A). Three thermocouple thermometer probes were buried onto the gastric serosa at the center, 1 cm and 2 cm from the center of the mark (Fig. 1B). The stomach was returned into the abdominal cavity, followed by incision closure. Then three minutes’ cryoablation was carried out and the temperature was monitored from the start of the cryoablation to the end of the operation.

#### The pathological examination of the gastric wall

Cryoablation tissues were harvested for general observation. And then the tissue was imbedded in paraffin followed by Hematoxylin-Eosin staining for microscopic observation of gastric structure and Masson staining for observation of collagen production and distribution.

### Statistics analysis

Summary data was expressed as mean and standard deviation. The Student’s T-Test was performed by using Stata/MP software (version 14) and differences between two groups with p value < 0.05 were considered statistically significant.

## Results

### *In vitro* experiments suggested 3 minutes’ cryoablation the optimum cryoablation duration

Different cryoablation durations were performed on the 3D cell culture dishes. The cells in the center area with temperature less than −40 °C were harvested for cell apoptosis analysis via a flow cytometry assay. One minute’, 3 minutes’ and 6 minutes’ cryoablation resulted in dramatically cell viability decrease (62.23%, 25.27%, 19.03% vs 80.77%, *p < 0.05), apoptosis cells increase (26.1%, 58.07%, 63.47% vs 7.26%, *p < 0.05) and dead cells increase (6.618%, 10.72%, 12.80% vs. 3.693%, *p < 0.05) compared with control. Three minutes’ cryoablation could cause markedly decrease living cells and increase apoptosis cells compared with 1 minute’s cryoablation (*p < 0.05), but similar effects with 6 minutes’ cryoablation without significant difference (Fig. S2). In consideration of increased perforation or bleeding risks when longer cryoablation duration was performed on stomach, 3 minutes’ duration was chosen as cryoablation persistent period.

### The effects of cryoablation on experimental animals

Seconds after the start of the cryoablation system, the low-temperature balloon could be fixed onto the targeted lesion via condensation, avoiding detachment caused by operation, pig’s breath or heartbeat or stomach peristalsis. Iceball gradually formed and expanded from the mucosal contact site to the surrounding. At 3 minutes, the iceball reached 1 cm in radius. After the cryoablation procedure ended, at 6 minutes the iceball gradually thawed, allowing the probe to be separated without adhesion to the mucosa. During the whole procedure, no active bleeding or acute perforation was observed. At 45 minutes, a repeated endoscope revealed hyperemia on the cryoablation site without signs of active bleeding or perforation (Fig. 1A).

**Figure 2:**
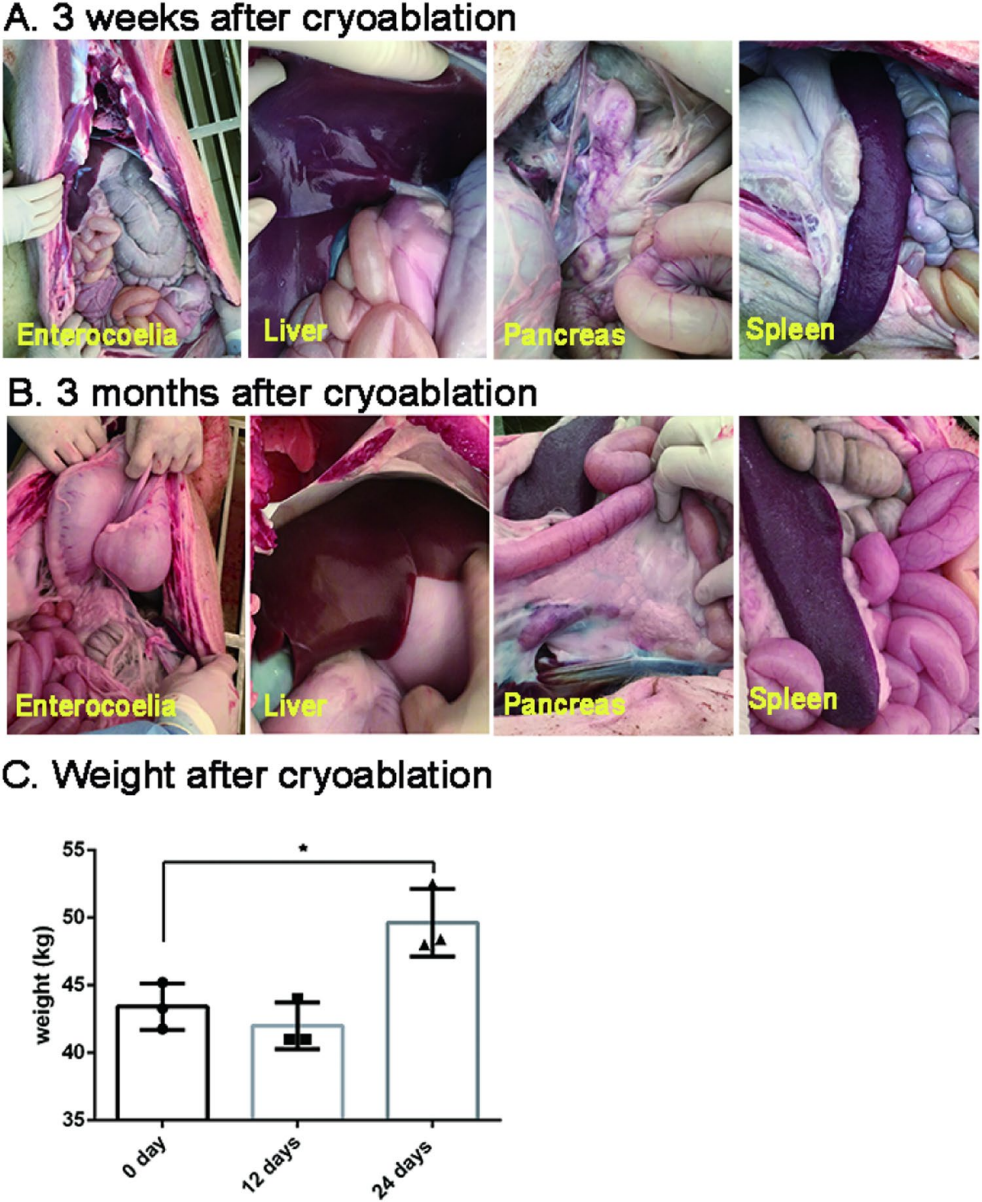
Influence of endoscopic gastric cryoablation on adjacent organs and porcine weight. (**A**) Appearance of adjacent organs 3 weeks after cryoablation. (**B**) Appearance of adjacent organs 3 months after cryoablation. (**C**) Changes of porcine weight after cryoablation. There was no significant weight decrease 12 days after the cryoablation. Increase in animal weight was observed in the experimental porcine 24 days after cryoablation compared to their preoperative status (*p < 0.05).

All nine pigs had maintained stable vital signs throughout the procedure. No acute perforation or active bleeding occurred during cryoablation. Re-examination by upper endoscopy confirmed these findings (Fig. 1A). No acute death caused by cryoablation occurred. During follow-up, no fever was found. Pigs in the subacute and chronic groups had normal intake without vomiting, hematemesis or melena. The preoperative weight of pigs in chronic groups was 43.42 ± 0.99 kg (n = 3). Twelve days later the weight was 42.00 ± 1.00 kg (n = 3), without remarkable decrease compared to preoperative weight. Twenty-four days later the weight was 49.63 ± 1.44 kg (n = 3) (Fig. 2C).

Dissection of pigs in the acute group suggested the organs adjacent to stomach were in normal condition without injury. As for pigs in subacute and chronic groups, no tissue adhesions were observed in the organs around the stomach, including left liver lobe, spleen, pancreas, transverse colon, mesentery and omentum, were all in normal condition (Fig. 2A,B).

### Temperature variations during the cryoablation operation

Upon cryoablation, the temperature at the center of the ablation site on the gastric serosal side decreased rapidly. At one minute of cryoablation, the temperature decreased to −17.2 °C, 11.7 °C, 34.5 °C at the center, 1 cm and 2 cm from the center, respectively. At two minutes, the temperature readout of the three sites were −50.3 °C, −18.7 °C, 31.4 °C, respectively. At three minutes, the temperature further decreased to −64.2 °C, −34.1 °C, and 26.1 °C at the three respective sites. After the cryoablation ended, the temperature gradually increased. The warming phase was consisted of one-minute rapid warming phase and following slow warming phase. Approximately 15 minutes later, the temperature at all three sites returned to primary level (Fig. 1C). The thickness of gastric antrum was 4.93 ± 0.80 mm.

### The histopathological founding of gastric wall

**Figure 3:**
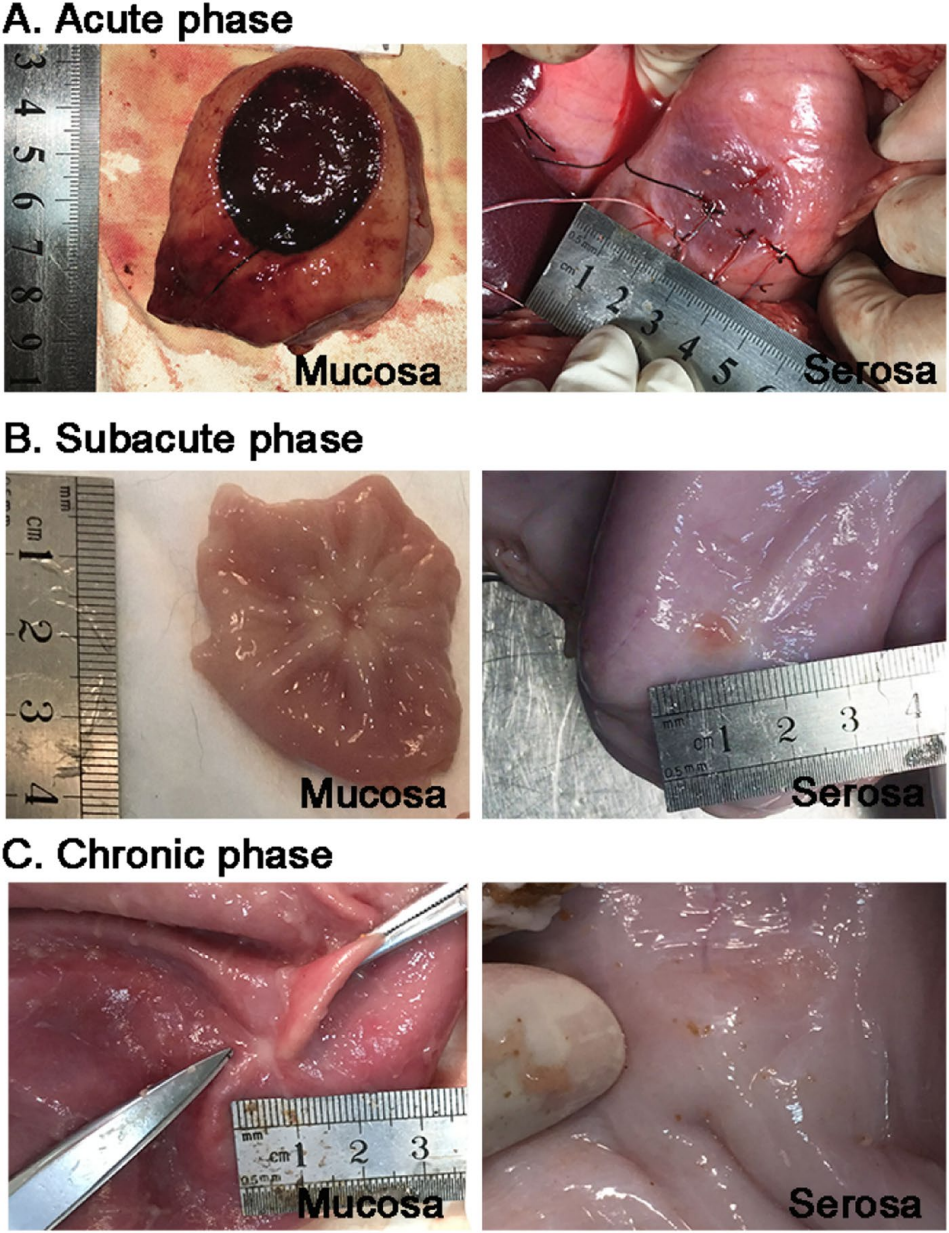
Gross morphological changes in the gastric mucosa and serosa immediately, 3 weeks and 3 months after cryoabaltion. (**A**) Acute change. On the gastric mucosal, a 2 cm radius “cryoburn” was observed. On the gastric serosa side, edema and hyperemia was observed without perforation in the acute group. (**B**) Subacute change. A healed wound with thickened surrounding plicas was seen in the subacute group. A circular orange mark was seen on the gastric serosa without local umbilication ridges. (**C**) Chronic change. A pale scar formed on the gastric mucosa 3 months later. No obvious cryoablation lesion was found on the gastric serosa in the chronic group.

After locating the cryoablation site in the acute phase, a 2 cm radius “cryoburn” on the mucosa and a 1.5 cm radius edema with hyperemia on the serosa (Fig. 3A) was observed. In subacute phase, the mucosa on the cryoablation site was smooth with a hollow surrounded by paled eminence composed of prominent gastric folds radially arranged from the center, while a smooth orange area was observed on the serosal side with no obvious local umbilication ridges (Fig. 3B). In chronic stage, a pale scar was formed on the cryoablation site of the gastric mucosa, while no obvious cryoablation lesion could be seen on the serosa side (Fig. 3C).

**Figure 4:**
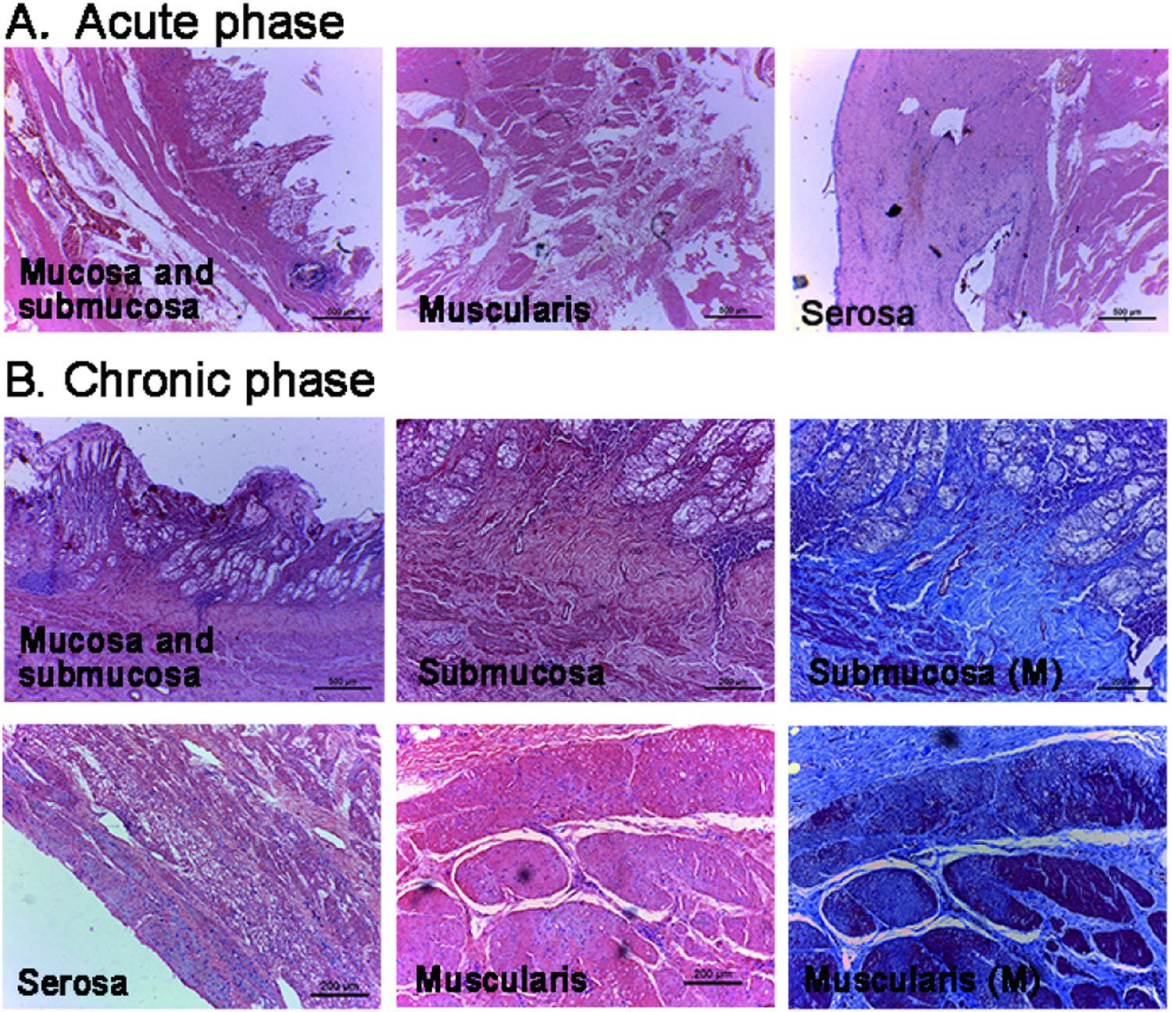
Pathologic changes of the gastric walls. (**A**) Acute phase (H&E, orig. mag. ×4). Sloughing of normal epithelium, cell burst and loss of normal gland structure, along with infiltration of inflammatory cells and obvious hyperemia were observed. Lamina propria and muscularis mucosa remained intact. Submucosa widened and was filled with leaking lymph fluid. Muscularis propria widened with partial disruption. Inflammatory cells infiltration in the serosa and exudates outside the serosa was observed. In the areas 1 cm to 2 cm from the center, partial mucosa remained, but normal structure was lost. The disruption of muscularis propria was less severe. An obvious juncture of cryoablation lesion and normal structure could be seen in the areas 2 cm to 3 cm from the center. (**B**) Chronic phase. The mucosa (H&E, orig. mag. ×4), the submucosa ands muscularis propria layer (H&E, orig. mag. ×10; Massion, orig. mag. ×10), the serosal layer (H&E, orig. mag. ×4). Chronic histopathology showed complete structure of mucosa and scar formation in the submucosa. The structure of muscularis propria and serosa was complete without exudates.

Histopathology revealed sloughing of normal epithelium, cell burst and loss of normal gland structure, within 1 cm radius from the center site, along with infiltration of inflammatory cells and obvious hyperemia. Lamina propria and muscularis mucosa remained intact. Submucosa widened and was filled with leaking lymph fluid and hyperemia. Muscularis propria widened with partial disruption. Infiltrating inflammatory cells and exudates were observed on the serosa side. Beyond 1 cm from the center, the disruption of the normal structure was less severe, but the injury can extend to 2 cm from the center (Fig. 4A). Subacute histopathology revealed re-epithelialization and continuous mucosa. Chronic histopathology showed complete structure of mucosa and scar formation in the submucosa. The structure of muscularis propria and serosa was complete without exudates (Fig. 4B).

## Discussions

This is first report on the *in vivo* observation of full-thickness cryoablation injury on gastric wall by using cryoablation device. Due to the technical restriction, the previous devices could not cause impairment on the whole layers of stomach, their deepest reaches less than half of gastric muscularis propria^3^. According to this study, three minutes’ cryoablation caused a transmural, 1 cm radius gastric lesion, cryoinjury was observed on the mucosa, submucosa and muscularis propria, with slight exudates on the serosal side.

During the whole ablation process, this device provided direct view of operation space. Unlike other spray cryoablation device, this one could allow the balloon to fixed onto the targeted lesion, enabling the controlled operation to improve the security and efficiency of cryoablation. Previous reports show that collagenous fibers and elastic fibers have high resistance to freezing, thus, cryotherapy would not damage large vessels which are rich in these fibers^8^. Pathological results in this study demonstrated intact blood vessels without active bleeding, which accorded with previous reports. Post-operative adhesions did not occur might due to the absence of bleeding or extravasation of fibrinogen-rich fluid from the injured surfaces and migration of inflammatory cells, that was observed in pathological section. The results suggested that the device was safe to be applied in the clinical transmural cryoablation of stomach.

This study simulated the real condition wherein the stomach was ablated in the abdomen, with simultaneous recording of treatment temperature. As an ablation mothed, cryoablation uses cold injury to kill cells. Various biological mechanisms have been reported to participate in cryoablative injury and occur in different zones of the cryolesion. They consisted of four main categories: direct cell injury, vascular injury and ischemia, apoptosis, and immunomodulation^1^. Several factors influence the effects of cryoablation. Reports show that the temperature necessary for cell lethality is between −40 °C and −20 °C^1,9^ However, few device has reported to attain this effective temperature throughout the whole gastric wall via endoscope. The balloon of the device could drive the temperature down to −120 °C. Three minutes cryoablation resulted in a circular field of 1 cm temperature lower than −30 °C on the gastric serosa (Fig. 1B). Histopathology revealed that cryoablation injuries were able to stretch to 2 cm from the center of cryoablation and extend to outmost layer of stomach. These results suggested that cryoablation could be applied for the treatment of gastric tumor with different depth of invasion. Previous study results indicate that slow thawing causes greater injury than rapid cooling and the repeat of freeze-thaw cycles can enhance lethal effect especially in the −20 to −30 °C temperature range^10^. According to our temperature-time curve (Fig. 1C), it took 50 seconds for temperature of the cryoablation center to increase from the −60°C to −20 °C, and took 110 seconds from −20 °C to −60 °C. To achieve better lethal effect on tissue, we could perform repeated cryoablation with about 50 seconds’ thawing and 110 seconds’ re-cryoablation. The temperature-time curve attained in this study gave us guidance on the following work.

This device could carry out convenient, accurate and effective ablation under endoscope, since the temperature and depth cryoablation attained was sufficient to produce lethal effect on gastric cancer cells that infiltrated all layers of stomach according to our study. In addition, cryoablation could not only kill tumor cells, but also activate immune system of the host against tumor^11^. Since 1980s, clinical researches continuously reported abscopal effects after the cryoablation of the primary tumor^12–15^. Furthermore, cryoablation of gastric tumor existed much less complication than other localized treatments. Lower incidence of bleeding, perforation and ill influence on the surrounding organs makes cryoablation more competitive than surgery or radiotherapy for localized treatment of gastric tumor. Therefore, multiple cryoablation of advanced gastric cancer is a potential application of the device which could reduce tumor load to improve the patients’ quality of life as well as activate the enduring anti-tumor effects.

## Supplementary information

Supplementary information

Supplementary information2

## References

[1] Chu KF, Dupuy DE (2014). Thermal Ablation of Tumours: Biological Mechanisms and Advances in Therapy. Nat. Rev. Cancer..

[2] Greenwald BD, Dumot JA, Horwhat JD, Lightdale CJ, Abrams JA (2010). Safety, Tolerability, and Efficacy of Endoscopic Low-Pressure Liquid Nitrogen Spray Cryotherapy in the Esophagus. Dis. Esophagus..

[3] Shin EJ (2012). Dose-Dependent Depth of Tissue Injury with Carbon Dioxide Cryotherapy in Porcine GI Tract. Gastrointest. Endosc..

[4] Cho S (2008). Endoscopic Cryotherapy for the Management of Gastric Antral Vascular Ectasia. Gastrointest. Endosc..

[5] Ribeiro A (2014). Depth of Injury Caused by Liquid Nitrogen Cryospray: Study of Human Patients Undergoing Planned Esophagectomy. Dig. Dis. Sci..

[6] Samarasena J, Chen C, Chin M, Chang K, Lee J (2017). Successful Closure of a Cryotherapy-Induced Bleeding Jejunal Perforation with the Over-The-Scope Clip System. Gastrointest. Endosc..

[7] Dumot JA (2009). An Open-Label, Prospective Trial of Cryospray Ablation for Barrett’s Esophagus High-Grade Dysplasia and Early Esophageal Cancer in High-Risk Patients. Gastrointest. Endosc..

[8] Eggstein S (2003). Hepatic Cryotherapy Involving the Vena Cava - Experimental Study in a Pig Liver Model. Eur. Surg. Res..

[9] Seifert, J. K. & Junginger, T. Cryotherapy for Liver Tumors: Current Status, Perspectives, Clinical Results, and Review of Literature. *Technology in Cancer Research & Treatment* **3**(2), 151–16 (2016). 10.1177/153303460400300208 15059021

[10] Gage AA, Baust J (1998). Mechanisms of Tissue Injury in Cryosurgery. Cryobiology..

[11] Sabel MS (2009). Cryo-Immunology: A Review of the Literature and Proposed Mechanisms for Stimulatory Versus Suppressive Immune Responses. Cryobiology..

[12] Soule E, Bandyk M, Matteo J (2018). Percutaneous Ablative Cryoimmunotherapy for Micrometastaic Abscopal Effect: No Complications. Cryobiology..

[13] Bonichon F (2015). Local Treatment of Metastases From Differentiated Thyroid Cancer. Ann. Endocrinol..

[14] Breitbart EW (1990). Cryosurgery in the Treatment of Cutaneous Malignant Melanoma. Clin. Dermatol..

[15] Cohen JK (2004). Cryosurgery of the Prostate: Techniques and Indications. Rev. Urol..

