# Supplementary material for: A novel endoscopic visible full-thickness cryoablation device on stomach
Source: Sci Rep. 2020 Mar 23;10:5203. doi: 10.1038/s41598-020-61595-x (PMC7090056; doi:10.1038/s41598-020-61595-x)
Supplement: Supplementary file 1 — Supplementary information [file 41598_2020_61595_MOESM1_ESM.pdf]

Supplementary Materials for

A novel endoscopic visible full-thickness cryoablation device on stomach

Wanwei Zheng[a#], Yao Liu[a#], Yujen Tseng[a], Jun Zhang[a], Wenshuai Li[a], Bangting Wang[a], Yida Pan[a], Jie Zhu[a], Zhongguang Luo[a*], Feifei Luo[a*], Jie Liu[a*]

[#]Authors Wanwei Zheng and Yao Liu contributed equally to the article.

a Department of Digestive Disease, Huashan Hospital, Fudan University, Shanghai 200040, China

Correspondence to：luozg8@126.com
feifeiluo@fudan.edu.cn
jieliu@fudan.edu.cn

This PDF file includes:

Figs. S1 to S2

Video information

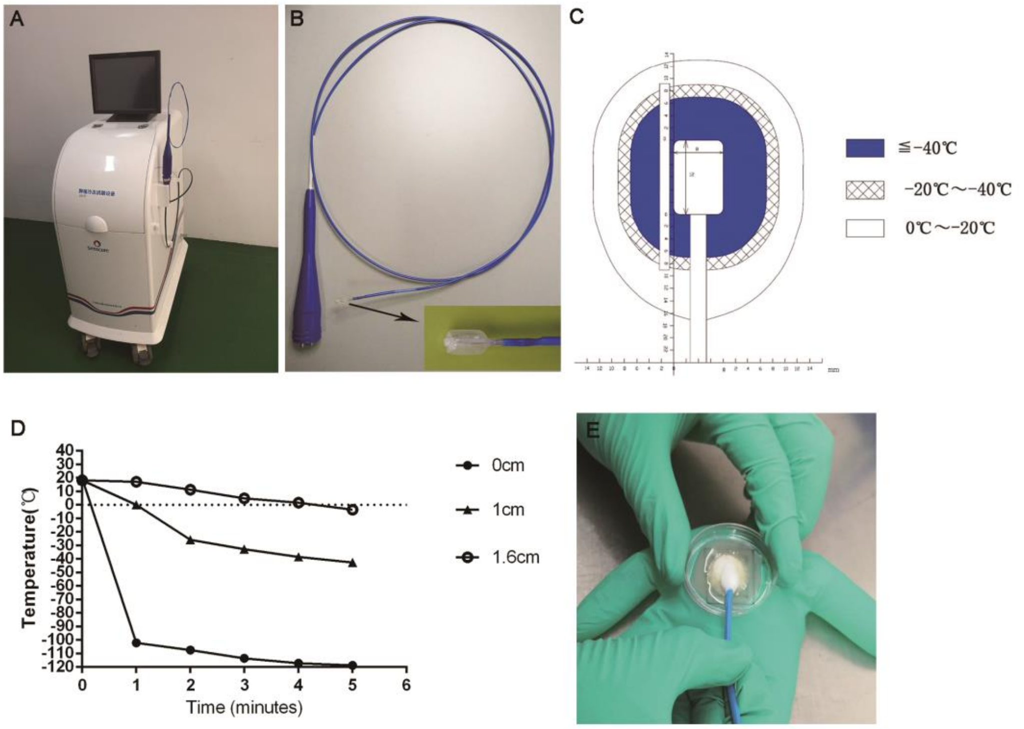

Figure. S1 (A) Cryoablation device. **(B)** Intraluminal tumor cryoablation catheter with distal balloon. **(C)** Temperature distribution diagram around the balloon. **(D)** Experiments in vitro showed that the temperature of the balloon surface could reach -120°C after 3 minutes in gelatin under the environment of 25 / 33% RH, forming a temperature field of 1cm in radius with temperature lower than -40°C, The temperature would not further obviously decrease after three minutes. **(E)** Experiments in vitro on 3D cell culture dish.

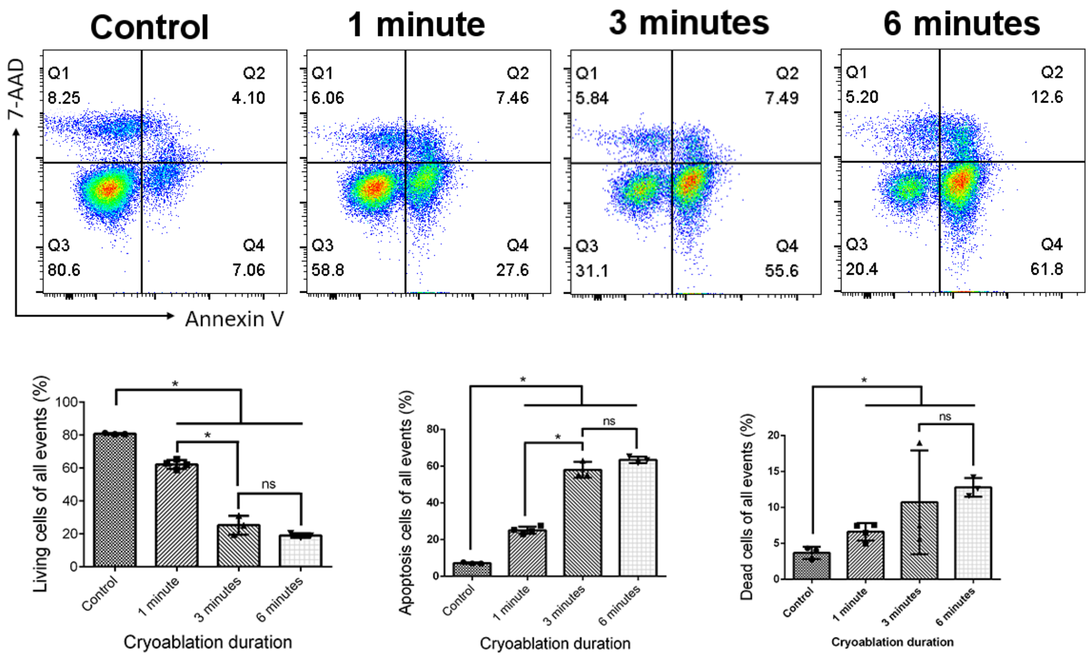

Figure. S2 Flow-cytometric analysis of living cells, apoptotic cells and dead cells after different cryoablation durations. One minute', 3 minutes' and 6 minutes' cryoablation resulted in dramatically cell viability decrease (62.23%, 25.27%, 19.03% vs. 80.77%, *p<0.05), apoptosis cells increase (26.1%, 58.07%, 63.47% vs. 7.26%, *p<0.05) and dead cells increase (6.618%, 10.72%, 12.80% vs. 3.693%, *p<0.05) compared with control. Three minutes' cryoablation could cause markedly decrease living cells and increase apoptosis cells compared with 1 minute's cryoablation (*p<0.05), but similar effects with 6 minutes' cryoablation without significant difference.

Video. S1 Procedure of cryoablation. It mainly contains five steps: (1) balloon positioning, (2) balloon inflation, (3) cryoablation start, (4) cryoablation end, (5) balloon separation.
